# The mediating role of sleep disorders in the relationship between breastfeeding and behavioral problems among 6- to 8-year-old children in Shanghai, China

**DOI:** 10.3389/fpubh.2025.1610810

**Published:** 2026-01-22

**Authors:** Yuli Hu, Zilu Shen, Chunsheng Wang, Xinyi Li, Jian Guan, Siqiong Jiang, Qunfeng Lu

**Affiliations:** 1 Department of Nursing, Shanghai Sixth People's Hospital Affiliated to Shanghai Jiao Tong University School of Medicine, Shanghai, China; 2 School of Nursing, Shanghai Jiao Tong University, Shanghai, China; 3 Department of Otorhinolaryngology Head and Neck Surgery, Shanghai Children's Hospital, School of Medicine, Shanghai Jiao Tong University, Shanghai, China; 4 School of Medicine, Huzhou University, Zhejiang, China; 5 Department of Otolaryngology, Shanghai Sixth People's Hospital Affiliated to Shanghai Jiao Tong University School of Medicine, Shanghai, China

**Keywords:** breastfeeding, childhood behavioral problems, a cross-sectional study, Children's Sleep Habits Questionnaire, children

## Abstract

**Background:**

Improving breastfeeding rates is a key target in the WHO Global Nutrition Targets 2025, yet the link between breastfeeding duration/exclusivity and childhood behavioral problems (BPs) remains controversial due to conflicting evidence. This study examined the role of sleep disorders in mediating the relationship between breastfeeding and BPs in children. Its aim was to provide a scientific basis for early intervention in children with BPs.

**Methods:**

This cross-sectional study was conducted across 34 elementary schools in Xuhui District, Shanghai, China, between September and December 2019. The valid data were collected from 11,319 primary school students aged 6–8 years. An online survey completed by their guardians was used to obtain demographic information and to score the Children's Sleep Habits Questionnaire (CSHQ) and Conners' Parent Rating Scale (CPRS). Descriptive statistics (frequencies and medians) were generated. Associations among variables were assessed using the Mann–Whitney *U* test, Spearman's rank correlation, and a path analysis (incorporating a bootstrap analysis to assess direct and indirect effects). CPRS scores were converted to z-scores using sex- and age-specific means and sleep disorders, with a z-score > 2 indicating abnormal behavior.

**Results:**

In our sample, the breastfeeding rate was 64.55% (*n* = 7,306/11,319). Controlling for age, gender, BMI z-score, parental age/education, breastfeeding was linked to higher overall sleep problem scores (CSHQ) and specific issues including bedtime resistance, sleep anxiety, and sleep-disordered breathing (all *p* < 0.05). At the same time, the results show that both breastfeeding and the 8 items of the CSHQ have direct and significant effects on the Learning problems, Impulsive—hyperactive, and Hyperactivity (all *p* < 0.05). Breastfeeding demonstrated significant total effects on multiple behavioral domains: conduct problems (β = 0.044, *p* = 0.025, indirect effect β = 0.030), Learning problems (β = 0.089, *p* < 0.001; direct β = 0.059, indirect β = 0.030), Psychosomatic problems (β = 0.072, *p* < 0.001; direct β = 0.048, indirect β = 0.024), Impulsive-hyperactive behaviors (β = 0.074, *p* < 0.001; direct β = 0.046, indirect β = 0.028), Anxiety (fully mediated, β = 0.054, *p* < 0.01, indirect β = 0.027), and Hyperactivity (β = 0.068, *p* < 0.01; direct β = 0.035, indirect β = 0.032). All 95% CIs for indirect effects excluded zero.

**Conclusion:**

Sleep disorders may mediate the relationship between the lack of breastfeeding and BPs in children 6–8 years of age, though our cross-sectional design precludes causal inference and parent-reported sleep disorders severity may bias true associations. Future studies should utilize longitudinal cohorts to explore whether sleep disorders is involved in the relationship between breastfeeding and behavioral problems.

## 1 Introduction

Maternal and child health has long been widely recognized by the international community as an important issue of global public health. In the World Health Organization's (WHO) Global Nutrition Targets 2025, improving breastfeeding is listed as one of the six nutrition goals, highlighting its key role in promoting healthy child development. Breastfeeding provides an ideal source of nutrition for infants (1). It has positive effects on children's sleep, such as promoting sleep and establishing circadian rhythms, and is also closely linked to children's cognitive development and behavioral performance (1, 2). However, there has been controversy in academia about the relationship between breastfeeding and child behavioral problems (BPs). While some studies have shown that breastfeeding may have a positive effect on children's cognitive development and behavioral performance (2–4), other studies and systematic reviews analyses have yielded inconsistent results regarding this association (5–7). This inconsistency in the relationship between breastfeeding and behavioral problems may stem from various factors, including study design, sample size, and the overlook of mediating variables like sleep disorders. These differing findings highlight the necessity for further research using large community samples and standardized instruments to assess behavioral issues, while considering important covariates.

The clinical urgency of resolving this controversy is underscored by the pervasive impacts of behavioral problems (BPs) on schoolchildren: Attention Deficit Hyperactivity Disorder (ADHD), anxiety, as well as detrimental effects on academic performance, social interactions, and long-term development, collectively affecting a significant portion of the student population and impairing their overall wellbeing (8–11). The childhood period is considered the optimal timeframe for early intervention regarding these behavioral patterns (BPs) (12). These findings emphasize the importance of early identification and intervention for childhood behavioral problems to promote their overall development.

Sleep disorders, which are common in childhood, exhibit independent links to both breastfeeding practices and behavioral problems (BPs). Studies have shown that sleep disturbances, such as insomnia, insufficient sleep, or poor sleep quality, can significantly impact children's emotional regulation, cognitive function, and behavioral performance (13). Insufficient sleep may lead to emotional dysregulation (like anxiety and depression), inattention, hyperactivity, and impulsive behavior, especially in children with attention deficit hyperactivity disorder (ADHD) (14, 15). Furthermore, sleep problems may exacerbate aggressive behavior and contribute to long-term behavioral issues that can persist into adolescence (16, 17). Notably, breastfeeding may reduce the incidence of sleep-disordered breathing (18), highlighting its potential protective role against such disorders in children. These evidences imply that sleep disorders may play a mediating role between breastfeeding and children's behavioral problems, although this mediating effect has not yet been verified. This finding enhances our clinical understanding of the relationships among breastfeeding, sleep disorders, and behavioral problems, underscoring the importance of promoting breastfeeding and effectively managing sleep and behavioral issues in children.

The main objectives of this cross-sectional study were: (1) to explore the association between breastfeeding and behavioral problems in children aged 6 to 8 years; (2) to elucidate the mediating effect of sleep disorders in the association between breastfeeding and behavioral problems.

## 2 Materials and methods

### 2.1 Study design and setting

This study constituted a cross-sectional investigation carried out in Xuhui District, Shanghai, China.

### 2.2 Subjects

We sent invitation letters to all 47 primary schools in the district. Thirteen schools declined to participate, leaving an initial sample of 26,827 students from 34 schools. From September to December 2019, teachers distributed consent forms and online questionnaires (with QR codes) to parents. A total of 19,033 parents agreed to participate, achieving a 71% consent rate. For this study, we selected 11,660 children in grades 1–3 (ages 6–8). Children with missing data (such as missing body mass index (BMI) scores, maternal age, or education level) were excluded, and 11,319 children were included in the valid data analysis. Teachers gave parents login codes to access the online survey, where they provided demographic information, medical history, and answers to the CSHQ and CPRS questionnaires. Height and weight data from recent physical exams were obtained from the Shanghai Education Commission's database. All parents provided informed consent online.

### 2.3 Materials and measures

#### 2.3.1 Demographic characteristics

The questionnaire included items designed to assess demographic characteristics, such as age, sex, and grade. WHO's Anthro Plus software was used to calculate age- and sex-adjusted BMI z-scores (standard deviation scores) (19).

#### 2.3.2 Breastfeeding and sleep disturbance

The feeding method of children, specifically breastfeeding, is based on data collected online.

“Based on the WHO guidelines (1), we ask guardians (i.e., parents, including either the father or mother acting independently, or both jointly) the following question: ‘Was your child exclusively breastfed for the first 6 months after birth? (Exclusive breastfeeding is defined as giving only breast milk, without adding any formula, solid foods, or other liquids.)' Options: Yes, No.”

Sleep disorders (SDs) were evaluated utilizing the Chinese adaptation of the Children's Sleep Habits Questionnaire (CSHQ), a commonly employed instrument for detecting sleep issues in children between the ages of 4 and 10 years (20). The CSHQ comprises 35 questions, with a total score exceeding 48 signifying the existence of overall sleep disturbances within the Chinese population (21). Furthermore, we examined eight distinct subscales of the CSHQ: bedtime resistance, sleep onset delay, sleep duration, sleep anxiety, night waking, parasomnias, sleep disordered breathing, and daytime sleepiness. Given the absence of established normative subscale scores for Chinese children in the targeted age range, a score that surpasses the population mean by more than two standard deviations is indicative of a clinically significant sleep disorder, as previously noted (21).

#### 2.3.3 Behavioral outcomes

The Conners' Parent Rating Scale (CPRS) was used to assess common behavioral problems. The CPRS consists of 48 items encompassing six factors: conduct problems, learning problems, psychosocial problems, impulsive—hyperactive, hyperactivity, and anxiety (22). Each item is rated on a 4-point scale ranging from 0 to 3, with higher scores indicating more pronounced behavioral issues (22). The CPRS scale was used only for non-commercial academic research.

To standardize the CPRS scores, the mean (M) and standard deviation (SD) values for different sexes and age groups on the Chinese version of the CPRS were used to convert the actual scores (X) of each child across various dimensions into z-scores using the formula Z = (X – M)/SD (23). In this study, a z-score > 2 was considered indicative of abnormal behavior (24).

#### 2.3.4 Data analysis

Data analysis was performed using SPSS version 24.0 and the PROCESS Procedure for SPSS version 4.1 (25). For categorical variables, descriptive statistics are presented as frequencies (percentages). Continuous variables are reported as Mean ± SD for data following a normal distribution and as median (interquartile range, P25–P75) for data without a normal distribution. Inter-group differences in the six dimensions of the CPRS questionnaire were analyzed using the Mann–Whitney *U* test. Spearman rank correlation analysis was conducted to explore the pairwise correlations among age, BMI z-score, parents' age and parents' education, total CSHQ score and the eight subdomains of the CSHQ, and the z-scores of the six CPRS dimensions. Model 4 in the PROCESS 4.1 program was used to evaluate the mediating effect of the total CSHQ score and the eight subdomains of the CSHQ on the relationship between breastfeeding and behavioral problems, as reflected in the z-scores of the CPRS dimensions. Based on the existing literature, age, sex, BMI z-score, parents' and parents' education were included as control variables. The bias-corrected percentile bootstrap method (with 1,000 bootstrap samples) was used to calculate 95% confidence intervals (CIs) for indirect effects. If the CI did not include 0, the effect was considered statistically significant; otherwise, it was deemed non-significant. When both direct and indirect effects were significant, the mediator variable was interpreted as exerting a partial mediation effect. If the indirect effect was significant but the direct effect was not, the mediator variable was considered to exert a full mediation effect. Cases with missing values were automatically excluded from the statistical analysis. All statistical tests were two-sided, with the significance level set at α = 0.05. A *p-value* ≤ 0.05 was considered statistically significant.

### 2.4 Ethical considerations

The research protocol adheres to the principles of the Declaration of Helsinki and was approved by the Ethics Committee of the Sixth People's Hospital of Shanghai Jiao Tong University (Grant No. 2018-008). Registration number: ChiCTR180001448.

## 3 Results

### 3.1 Demographics and clinical characteristics

For this study, we initially selected 11,660 children in grades 1–3 (ages 6–8) as potential participants. Three hundred and forty one children were excluded due to missing data like BMI scores, maternal age, or education level, etc. Finally, 11,319 children were included in the valid data analysis as actual participants. In our sample, the breastfeeding rate was 64.54% (*n* =7,306 out of 11,319). The mean age of primary school participants was 6.96 years (SD = 0.799). The number of boys was 6,002, accounting for 53%; the number of girls was 5,317, accounting for 47%. Additional details can be found in Tables 1, 2.

**Table 1:** Characteristics of the participants (*n* = 11,319).

**Characteristic**	**M (P25, P75)/ Mean ±SD/n (%)**
Age (year)	6.96 ± 0.799
Boy	6,002 (53.03%)
BMI z-score	0.32 (−0.45, 1.29)
Breastfeeding	7,306 (64.55%)
**Maternal educational level**	
Below High School	1,924 (17.00%)
Junior College/Diploma	2,898 (25.60%)
Bachelor's Degree	6,104 (53.93%)
Graduate Degree /Postgraduate	393 (3.47%)
**Father educational level**	
High school or below	2,623 (23.17%)
Junior college	2,810 (24.83%)
Graduate	5,576 (49.26%)
Postgraduate	310 (2.74%)
CSHQ total score abnormal	2,780 (24.56%)
CSHQ total score	48 (43.53)
Bedtime resistance	9 (8.12)
Sleep onset delay	1 (1.1)
Sleep duration	5 (3.6)
Sleep anxiety	7 (5.8)
Night wakings	3 (3.3)
Parasomnias	8 (7.9)
Sleep-disordered breathing	3 (3.3)
Daytime sleepiness	10 (9.12)
Conduct problems	605 (5.34%)
Learning problems	779 (6.88%)
Psychosomatic problems	429 (3.79%)
Impulsive-hyperactive	810 (7.16%)
Anxiety	740 (6.54%)
Hyperactivity	873 (7.71%)

Mean ± SD; CPRS, Conners Parent Rating Scale questionnaire; BMI, body mass index; CSHQ, Children's Sleep Habits Questionnaire; for the CSHQ total score, a score of 48 or higher is considered abnormal.

**Table 2:** Inter-group comparison based on dichotomous variable groupings of breastfeeding status, the eight dimensions of CSHQ and the six dimensions of CPRS (*n* = 11,319).

**Comparison Dimension**	**With breastfeeding, M (P25, P75)**	**Without breastfeeding M (P25, P75)**	Z^*^	***p***
CSHQ total score	48 (43, 53)	48 (43, 54)	−3.709	<0.001
Bedtime resistance	9 (8, 12)	10 (8, 12)	−3.189	0.001
Sleep onset delay	1 (1, 1)	1 (1, 1)	−2.313	0.021
Sleep duration	5 (3, 6)	5 (4, 6)	−1.712	0.087
Sleep anxiety	7 (5, 8)	7 (5, 8)	−2.299	0.022
Night waking's	3 (3, 3)	3 (3, 3)	−0.051	0.959
Parasomnias	8 (7, 9)	8 (7, 9)	−3.499	<0.001
Sleep-disordered breathing	3 (3, 3)	3 (3, 3)	−4.086	<0.001
Daytime sleepiness	10 (9, 12)	10 (9, 12)	−1.471	0.141
Conduct problems score	−0.310 (−0.885, 0.552)	−0.240 (−0.885, 0.552)	−3.072	0.002
Learning problems score	−0.022 (−0.735, 0.796)	−0.022 (−0.735, 0.931)	−5.244	<0.001
Psychosomatic problems score	−0.652 (−0.654, 0.217)	−0.652 (−0.654, 0.217)	−2.964	0.003
Impulsive - hyperactive	−0.170 (−0.911, 0.894)	−0.170 (−0.911, 0.894)	−3.889	<0.001
Anxiety score	−0.219 (−0.895, 0.563)	−0.219 (−0.895, 0.563)	−2.956	0.003
Hyperactivity score	−0.294 (−0.882, 0.737)	−0.053 (−0.842, 0.737)	−4.276	<0.001

^*^Mann-Whitney *U* Test.

### 3.2 Bivariate correlation analyses

Table 3 shows the correlation between the control variables and the dependent variable, as well as the mediator variable. There is a significant correlation between children's age and BMI z-score, parents' age, CSHQ total score, Bedtime Resistance, Sleep Onset Delay, Sleep Duration, Sleep Anxiety, Night Wakings, Daytime Sleepiness, Learning problems, and Psychosomatic problems, with *p* < 0.05. The other details can be found in Table 3.

**Table 3:** Spearman correlation values for all relationships between variables (*n* = 11,319).

**Variables**	**1**	**2**	**3**	**4**	**5**	**6**	**7**	**8**	**9**	**10**	**11**	**12**	**13**	**14**	**15**	**16**	**17**	**18**	**19**	**20**	**21**
1. Age	–																				
2. BMI z _score	0.068^**^	–																			
3. Father's age	0.144^**^	−0.009	–																		
4. Father's education	0.01	0.011	−0.088^**^	–																	
5. Mother's age	0.144^**^	−0.014	0.719^**^	−0.080^**^	–																
6. Mother's education	0.009	0.005	−0.051^**^	0.326^**^	−0.082^**^	–															
7. CSHQ total score	−0.054^**^	−0.036^**^	−0.035^**^	0.006	−0.035^**^	0.011	–														
8. Bedtime resistance	−0.131^**^	−0.025^**^	−0.038^**^	−0.001	−0.038^**^	0.012	0.764^**^	–													
9. Sleep onset delay	−0.037^**^	−0.016	−0.034^**^	0.027^**^	−0.042^**^	0.007	0.325^**^	0.191^**^	–												
10. Sleep duration	0.073^**^	−0.032^**^	0.027^**^	0.007	0.036^**^	0.018	0.513^**^	0.186^**^	0.262^**^	–											
11. Sleep anxiety	−0.089^**^	−0.015	–.032^**^	−0.003	−0.033^**^	0.006	0.773^**^	0.779^**^	0.165^**^	0.164^**^	–										
12. Night waking's	–.029^**^	0.004	–.020^*^	0.01	−0.012	0.004	0.352^**^	0.137^**^	0.112^**^	0.133^**^	0.196^**^	–									
13. Parasomnias	−0.008	−0.011	–.040^**^	0.018	−0.052^**^	0.015	0.524^**^	0.214^**^	0.137^**^	0.184^**^	0.269^**^	0.284^**^	–								
14. Sleep disordered breathing	0.012	0.054^**^	−0.047^**^	0.030^**^	−0.055^**^	0.025^**^	0.288^**^	0.111^**^	0.072^**^	0.118^**^	0.128^**^	0.147^**^	0.275^**^	–							
15. Daytime sleepiness	0.020^*^	−0.054^**^	−0.006	−0.012	−0.008	−0.011	0.644^**^	0.226^**^	0.163^**^	0.346^**^	0.285^**^	0.192^**^	0.309^**^	0.179^**^	–						
16. Conduct problems	0.015	−0.01	−0.020^*^	−0.004	−0.019^*^	−0.014	0.417^**^	0.212^**^	0.158^**^	0.272^**^	0.231^**^	0.174^**^	0.287^**^	0.155^**^	0.401^**^	–					
17. Learning problems	0.071^**^	−0.008	–.038^**^	0.037^**^	−0.052^**^	0.021^*^	0.412^**^	0.201^**^	0.164^**^	0.293^**^	0.225^**^	0.160^**^	0.272^**^	0.167^**^	0.394^**^	0.696^**^	–				
18. Psychosomatic problems	0.040^**^	−0.149^**^	0.028^**^	0.000	0.013	0.006	0.272^**^	0.142^**^	0.099^**^	0.191^**^	0.160^**^	0.105^**^	0.181^**^	0.087^**^	0.259^**^	0.335^**^	0.342^**^	–			
19. Impulsive– hyperactive	−0.01	0.006	–.065^**^	0.029^**^	−0.068^**^	0.013	0.370^**^	0.180^**^	0.145^**^	0.239^**^	0.203^**^	0.164^**^	0.298^**^	0.160^**^	0.330^**^	0.729^**^	0.661^**^	0.279^**^	–		
20. Anxiety	0.005	−0.063^**^	−0.028^**^	0.015	−0.037^**^	0.009	0.356^**^	0.209^**^	0.118^**^	0.195^**^	0.251^**^	0.140^**^	0.233^**^	0.133^**^	0.302^**^	0.495^**^	0.475^**^	0.298^**^	0.387^**^	–	
21. Hyperactivity	0.015	−0.013	−0.049^**^	0.032^**^	−0.056^**^	0.016	0.442^**^	0.221^**^	0.172^**^	0.294^**^	0.244^**^	0.187^**^	0.317^**^	0.177^**^	0.408^**^	0.816^**^	0.887^**^	0.342^**^	0.872^**^	0.498^**^	

Spearman's Rho: ^**^ Significance at p <0.01; ^*^Significance at p <0.05.

1 = Ages; 2 = Father's Age; 3 = Father's education; 4 = Mother's Age; 5 = Mother's education; 1 = Age; 2 = BMI z _score; 3 = Father's Age; 4 = Father's education; 5 = Mother's Age; 6 = Mother's education; 7 = CSHQ total score; 8 = Bedtime Resistance; 9 = Sleep onset Delay; 10 = Sleep Duration; 11 = Sleep Anxiety; 12 = Night Waking's; 13 = Parasomnias; 14 = Sleep Disordered Breathing; 15 = Daytime Sleepiness; 16 = Conduct problems; 17 = Learning problems; 18 = Psychosomatic problems; 19 = Impulsive– hyperactive; 20 = Anxiety; 21 = Hyperactivity.

### 3.3 Mediation models

#### 3.3.1 Definitions of direct, indirect, and total effects

In the context of this study, the direct effect refers to the direct influence of the independent variable (breastfeeding) on the dependent variable (scores on the six dimensions of the CPRS questionnaire) without going through the mediator (the total CSHQ score and its subscale scores). The indirect effect represents the influence of breastfeeding on the CPRS scores that is transmitted via the total CSHQ score. The total effect is the combined influence of breastfeeding on the CPRS scores, encompassing both the direct and indirect effects.

#### 3.3.2 The relationship between breastfeeding, the full CSHQ score and its subscale scores

Table 4 shows the relationship between independent Variable (breastfeeding) and Mediator Variable (the total CSHQ score and its subscales). After controlling for the effects of age, gender, BMI z- score, parents' age, and parents' education, breastfeeding was found to be positively associated with the total CSHQ score (*B* = 0.549, SE = 0.411, *p* < 0.001) as well as with bedtime resistance, sleep onset delay, sleep anxiety, parasomnias, and sleep-disordered breathing (all *p* < 0.05) (Table 4).

**Table 4:** Analysis of the impact of breastfeeding on the total score and dimensions of CSHQ.

**Variables**	**M = Total CSHQ score**	**Subscale1: Bedtime resistance**	**Subscale 2: Sleep onset delay**	**Subscale 3: Sleep duration**	**Subscale 4: Sleep anxiety**	**Subscale 5: Night wakings**	**Subscale 6: Parasomnias**	**Subscale 7: Sleep disordered breathing**	**Subscale 8: daytime sleepiness**
**Path**	**B(SE)p**	**B(SE)p**	**B(SE)p**	**B(SE)p**	**B(SE)p**	**B(SE)p**	**B(SE)p**	**B(SE)p**	**B(SE)p**
constant	51.762 (0.958) ^**^	12.338 (0.335) ^**^	1.472 (0.068) ^**^	3.365 (0.208) ^**^	8.147 (0.254) ^**^	3.797 (0.106) ^**^	8.958 (0.2) ^**^	3.363 (0.070) ^**^	10.321 (0.299) ^**^
X = Breastfeeding	0.549 (0.145) ^**^	0.176 (0.051) ^**^	0.025 (0.010) 0.016	0.050 (0.032) ^**^	0.108(0.038) ^**^	−0.007 (0.016) 0.650	0.091 (0.03) ^**^	0.029 (0.011) ^**^	0.077 (0.045) 0.089
*R*	0.077	0.141	0.058	0.094	0.089	0.054	0.062	0.098	0.079
R2	0.006	0.020	0.003	0.009	0.008	0.003	0.004	0.010	0.006
*F*	8.439^**^	28.552^**^	4.759^**^	12.636^**^	11.343^**^	4.156^**^	5.377^**^	13.675^**^	8.975^**^

Control variables: age, Gender, BMI z-score, educational levels of both parents and parental ages; X: independent variable; M: mediator variable Total CSHQ Score, Bedtime Resistance, Sleep Onset Delay, Sleep Duration, Sleep Anxiety, Night Waking's, Parasomnias, Sleep Disordered Breathing, and Daytime Sleepiness.

^**^p <0.01.

#### 3.3.3 Direct effects

After controlling for the effects of age, gender, BMI z- score, parents' age, and parents' education, the results show that both breastfeeding and the 8 items of the CSHQ have direct and significant effects on the Learning problems, Impulsive—hyperactive, and Hyperactivity (all *p* < 0.05), the details of other variables can be found in Table 5. The direct effects of the control variables, breastfeeding, and the total score of the Children's CSHQ on the six dimensions of the CPRS are provided in Table 6.

**Table 5:** Direct effects of breastfeeding and CSHQ subscales on CPRS dimensions after controlling for covariates.

**Variables**	**Y1: Conduct problems**		**Y2: Learning problems**		**Y3: Psychosomatic problems**		**Y4: Impulsive-hyperactive**		**Y5: Anxiety**		**Y6: Hyperactivity**	
	**B (SE)**	**95% CI**	**B (SE)**	**95% CI**	**B (SE)**	**95% CI**	**B (SE)**	**95% CI**	**B (SE)**	**95% CI**	**B (SE)**	**95% CI**
Constant	−3.700 (0.156)	−4.007 – −3.393	−3.828 (0.158)	−4.137 – −3.519	−3.105 (0.140)	−3.379 – −2.832	−2.995 (0.173)	−3.334 – −2.657	−2.824 (0.149)	−3.116 –−2.531	−3.845 (0.161)	−4.160 – −3.530
X= Breastfeeding	0.017 (0.020)	−0.023 – 0.056	0.069 (0.020)	0.030 – 0.109	0.047 (0.018)	0.012 – 0.082	0.057 (0.022)	0.014 – 0.101	0.029 (0.019)	−0.009 – 0.066	0.043 (0.021)	0.002 – 0.083
M1: Bedtime resistance	0.029 (0.006)	0.018 – 0.040	0.021 (0.006)	0.010 – 0.032	0.004 (0.005)	−0.006 – 0.014	0.017 (0.006)	0.004 – 0.029	0.009 (0.006)	−0.002 – 0.019	0.026 (0.006)	0.015 – 0.038
M 2: Sleep onset delay	0.104 (0.019)	0.066 – 0.142	0.112 (0.019)	0.074 – 0.150	0.064 (0.017)	0.030 – 0.098	0.104 (0.021)	0.062 – 0.145	0.069 (0.018)	0.033 – 0.105	0.115 (0.020)	0.076 – 0.154
M 3: Sleep duration	0.086 (0.007)	0.073 – 0.099	0.103 (0.007)	0.090 – 0.116	0.055 (0.006)	0.044 – 0.067	0.085 (0.007)	0.071 – 0.099	0.050 (0.006)	0.038 – 0.063	0.099 (0.007)	0.085 – 0.112
M 4: Sleep anxiety	0.007 (0.008)	−0.008 – 0.023	0.023 (0.008)	0.008 – 0.039	0.031 (0.007)	0.018 – 0.045	0.020 (0.009)	0.003 – 0.037	0.071 (0.007)	0.057 – 0.086	0.018 (0.008)	0.003 – 0.034
M 5: Night wakings	0.055 (0.013)	0.030 – 0.080	0.029 (0.013)	0.004 – 0.054	0.043 (0.011)	0.020 – 0.065	0.041 (0.014)	0.013 – 0.068	0.037 (0.012)	0.013 – 0.060	0.051 (0.013)	0.025 – 0.077
M 6: Parasomnias	0.102 (0.007)	0.087 – 0.116	0.086 (0.007)	0.072 – 0.101	0.066 (0.006)	0.054 – 0.079	0.139 (0.008)	0.124 – 0.155	0.078 (0.007)	0.065 – 0.091	0.128 (0.007)	0.113 – 0.142
M 7: Sleep disordered breathing	0.070 (0.019)	0.032 – 0.108	0.067 (0.019)	0.029 – 0.105	0.120 (0.017)	0.087 – 0.154	0.069 (0.021)	0.028 – 0.111	0.070 (0.018)	0.034 – 0.106	0.082 (0.020)	0.044 – 0.121
M 8: Daytime sleepiness	0.137 (0.005)	0.128 – 0.146	0.142 (0.005)	0.132 – 0.151	0.068 (0.004)	0.059 – 0.076	0.116 (0.005)	0.106 – 0.126	0.088 (0.005)	0.079 – 0.097	0.140 (0.005)	0.131 – 0.150
R	0.470		0.484		0.359		0.432		0.407		0.498	
R^2^	0.221		0.234		0.129		0.186		0.166		0.248	
F	200.093		215.686		104.488		161.778		140.129		232.952	
P	<0.0001		<0.0001		<0.0001		<0.0001		<0.0001		<0.0001	

Control variables: age, Gender, BMI-Z-score, educational levels of both parents and parental ages; X, independent variable; M, mediator variable; Y, the dependent variable; SE, Standard Error; CI, Confidence Intervals.

**Table 6:** Direct effects of breastfeeding and CSHQ total score on CPRS dimensions after controlling for covariates.

**Variables**	**Y1: Conduct problems**		**Y2: Learning problems**		**Y3: Psychosomatic problems**		**Y4: Impulsive-hyperactive**		**Y5: Anxiety**		**Y6: Hyperactivity**	
	**B(SE)**	**95% CI**	**B(SE)**	**95% CI**	**B(SE)**	**95% CI**	**B(SE)**	**95% CI**	**B(SE)**	**95% CI**	**B(SE)**	**95% CI**
Constant	−3.495 (0.152)	−3.794 – −3.197	−3.779 (0.154)	−4.080 – −3.478	−2.838 (0.134)	−3.100 – −2.575	−2.708 (0.167)	−3.036 – −2.380	−2.731 (0.143)	−3.010 – −2.451	−3.599 (0.157)	−3.906 – −3.292
X= Breastfeeding	0.014 (0.021)	−0.026 – 0.055	0.067 (0.021)	0.026 – 0.108	0.047 (0.018)	0.011 – 0.082	0.057 (0.023)	0.013 – 0.102	0.027 (0.019)	−0.010 – 0.065	0.041 (0.021)	0.000 – 0.083
M=– Total CSHQ Score	0.066 (0.001)	0.064 – 0.069	0.067 (0.001)	0.065 – 0.070	0.042 (0.001)	0.040 – 0.044	0.065 (0.001)	0.062 – 0.068	0.055 (0.001)	0.053 – 0.057	0.073 (0.001)	0.070 – 0.076
R	0.425		0.439		0.332		0.390		0.391		0.077	
R^2^	0.181		0.193		0.110		0.152		0.153		0.454	
F	277.074		300.461		155.908		226.040		227.35		325.975	
p	<0.0001		<0.0001		<0.0001		<0.0001		<0.0001		<0.0001	

M, Mediator; Y, Outcome variable; SE, Standard Error; CI, Confidence Intervals. Control variables= age, Gender, BMI-Z-score, educational levels of both parents and parental ages. X, Breastfeeding; M, Total CSHQ Score, Bedtime Resistance, Sleep Onset Delay, Sleep Duration, Sleep Anxiety, Night Wakings, Parasomnias, Sleep Disordered Breathing, and Daytime Sleepiness.

#### 3.3.4 Mediated effects

Table 7 shows that breastfeeding had a statistically significant overall effect on Conduct problems (β = 0.044, *p* = 0.025), with a total indirect effect of [β = 0.030, 95% CI (0.012–0.055)]. It also had a statistically significant overall effect on Learning problems (β = 0.089, *p* < 0.001), comprising a direct effect (β = 0.059, *p* < 0.001) and a total indirect effect [β = 0.030, 95% CI (0.014–0.056)]. Breastfeeding exhibited a statistically significant total effect on Psychosomatic problems (β = 0.072, *p* < 0.001), including a direct effect (β = 0.048, *p* < 0.001) and a total indirect effect [β = 0.024, 95% CI (0.009–0.036)]. Similarly, it had significant total effects on Impulsive-hyperactive behaviors (β = 0.074, *p* < 0.001), encompassing a direct effect (β = 0.046, *p* < 0.05) and a total indirect effect [β = 0.028, 95% CI (0.015–0.056)]. Additionally, breastfeeding demonstrated statistically significant total effects on Anxiety (β = 0.054, *p* < 0.01), entirely mediated by an indirect effect [β = 0.027, 95% CI (0.013–0.046)], and on Hyperactivity (β = 0.068, *p* < 0.01), with both a total effect (β = 0.035, *p* < 0.05) and a total indirect effect [β = 0.032, 95% CI (0.015–0.061)]. Details of other variables can be found in Supplementary Table S1.

**Table 7:** The mediating role of CSHQ dimensions in the scores of six dimensions of the CPRS among breastfeeding infants.

**Y: Variable**	**Effect term**	**B (SE)**	95%CI^*^	**β**	***p***
**Y1: Conduct** **problems**	Total effect (c)	0.051 (0.023)	0.006–0.095	0.044	0.025
	Direct effect (c')	0.017 (0.02)	−0.023–0.056	0.014	0.411
	Total indirect effect	0.034 (0.011)	0.012 −0.055	0.030	
	M1 Bedtime resistance	0.005 (0.002)	0.002 −0.009	0.004	
	M2 Sleep onset delay	0.003 (0.001)	0.001 −0.005	0.002	
	M3 Sleep duration	0.004 (0.003)	−0.001 −0.010	0.004	
	M4 Sleep anxiety	0.001 (0.001)	−0.001 −0.003	0.001	
	M5 Night waking's	0.000 (0.001)	−0.002 −0.001	<0.001	
	M6 Parasomnias	0.009 (0.003)	0.003–0.016	0.008	
	M7 Sleep disordered breathing	0.002 (0.001)	0.000 −0.005	0.002	
	M8 Daytime sleepiness	0.011 (0.006)	−0.002 −0.023	0.009	
**Y2:Learning problems**	Total effect (c)	0.104 (0.023)	0.059–0.149	0.089	<0.001
	Direct effect (c')	0.069 (0.020)	0.030–0.109	0.059	0.001
	Total indirect effect	0.035 (0.011)	0.014–0.056	0.030	
	M1 Bedtime resistance	0.004 (0.002)	0.001–0.007	0.003	
	M2 Sleep onset delay	0.003 (0.001)	0.000–0.005	0.002	
	M3 Sleep duration	0.005 (0.003)	−0.001 −0.012	0.004	
	M4 Sleep anxiety	0.003 (0.001)	0.000–0.005	0.002	
	M5 Night waking's	0.000 (0.001)	−0.001 −0.001	<0.001	
	M6 Parasomnias	0.008 (0.003)	0.003–0.014	0.007	
	M7 Sleep disordered breathing	0.002 (0.001)	0.000–0.004	0.002	
	M8 Daytime sleepiness	0.011 (0.006)	−0.003– 0.024	0.009	
**Y3:Psychosomatic problems**	Total effect (c)	0.070 (0.019)	0.032–0.107	0.072	<0.001
	Direct effect (c')	0.047 (0.018)	0.012–0.082	0.048	0.009
	Total indirect effect	0.023 (0.007)	0.009–0.036	0.024	
	M1 Bedtime resistance	0.001 (0.001)	−0.001 −0.003	0.001	
	M2 Sleep onset delay	0.002 (0.001)	0.000–0.004	0.002	
	M3 Sleep duration	0.003 (0.002)	−0.001 −0.006	0.003	
	M4 Sleep anxiety	0.003 (0.002)	0.001–0.007	0.003	
	M5 Night waking's	0.000 (0.001)	−0.002– 0.001	<0.001	
	M6 Parasomnias	0.006 (0.002)	0.002–0.011	0.006	
	M7 Sleep disordered breathing	0.004 (0.001)	0.001–0.007	0.004	
	M8 Daytime sleepiness	0.005 (0.003)	−0.001 −0.012	0.005	
**Y4:** **Impulsive- hyperactive**	Total effect (c)	0.093 (0.024)	0.045–0.141	0.074	<0.001
	Direct effect (c')	0.057 (0.022)	0.014–0.101	0.046	0.010
	Total indirect effect	0.035 (0.010)	0.015–0.056	0.028	
	M1 Bedtime resistance	0.003 (0.002)	0.000–0.007	0.002	
	M2 Sleep onset delay	0.003 (0.001)	0.001–0.005	0.002	
	M3 Sleep duration	0.004 (0.003)	−0.001 −0.009	0.003	
	M4 Sleep anxiety	0.002 (0.001)	0.000–0.005	0.002	
	M5 Night waking's	0.000 (0.001)	−0.002 −0.001	<0.001	
	M6 Parasomnias	0.013 (0.004)	0.005–0.022	0.010	
	M7 Sleep disordered breathing	0.002 (0.001)	0.000–0.005	0.002	
	M8 Daytime sleepiness	0.009 (0.005)	−0.001 −0.019	0.007	
**Y5:** **Anxiety**	Total effect (c)	0.058 (0.021)	0.017–0.099	0.054	0.006
	Direct effect (c')	0.029 (0.019)	−0.009 −0.066	0.027	0.136
	Total indirect effect	0.029 (0.008)	0.013–0.046	0.027	
	M1 Bedtime resistance	0.002 (0.001)	−0.001 −0.004	0.001	
	M2 Sleep onset delay	0.002 (0.001)	0.000–0.004	0.002	
	M3 Sleep duration	0.002 (0.002)	−0.001 −0.006	0.002	
	M4 Sleep anxiety	0.008 (0.003)	0.002–0.013	0.007	
	M5 Night waking's	0.000 (0.001)	−0.002 −0.001	<0.001	
	M6 Parasomnias	0.007 (0.002)	0.002–0.012	0.007	
	M7 Sleep disordered breathing	0.002 (0.001)	0.000–0.005	0.002	
	M8 Daytime Sleepiness	0.007 (0.004)	−0.002 −0.015	0.006	
**Y6:** **Hyperactivity**	Total effect (c)	0.081 (0.024)	0.035–0.128	0.068	0.001
	Direct effect (c')	0.043 (0.021)	0.002–0.083	0.035	0.039
	Total indirect effect	0.039 (0.012)	0.015–0.061	0.032	
	M1 Bedtime resistance	0.005 (0.002)	0.002–0.009	0.004	
	M2 Sleep onset delay	0.003 (0.001)	0.001–0.006	0.002	
	M3Sleep duration	0.005 (0.003)	−0.001 −0.011	0.004	
	M4 Sleep anxiety	0.002 (0.001)	0.000–0.005	0.002	
	M5 Night waking's	0.000 (0.001)	−0.002 −0.001	<0.001	
	M6 Parasomnias	0.012 (0.004)	0.004–0.019	0.010	
	M7 Sleep disordered breathing	0.002 (0.001)	0.000–0.005	0.002	
	M8 Daytime sleepiness	0.011 (0.006)	−0.002 −0.023	0.009	

^*^The confidence interval (CI) for the total direct effect was calculated using the Bootstrap method; CPRS questionnaires: Conners Parent Rating Scale.

X = breastfeeding; M = CSHQ dimensions (M1-8); Y = CPRS behavioral dimensions (Y1-6).

## 4 Discussion

The large cross-sectional study found that self-reported lack of breastfeeding, compared to breastfeeding, is associated with an increased risk of behavioral problems in 6–8-year-old children.

After controlling for the effects of age, gender, BMI z- score, parents' age, and parents' education, the results show that breastfeeding partially or fully mediates children' behavioral problems. This finding provides an intervenable target pathway for the early prevention and control of behavioral problems in children. It is important to emphasize that this was a cross-sectional study, and the mediating effect observed in the research findings merely indicated a statistical association, without implying any causal relationships among the variables.

**Figure 1:**
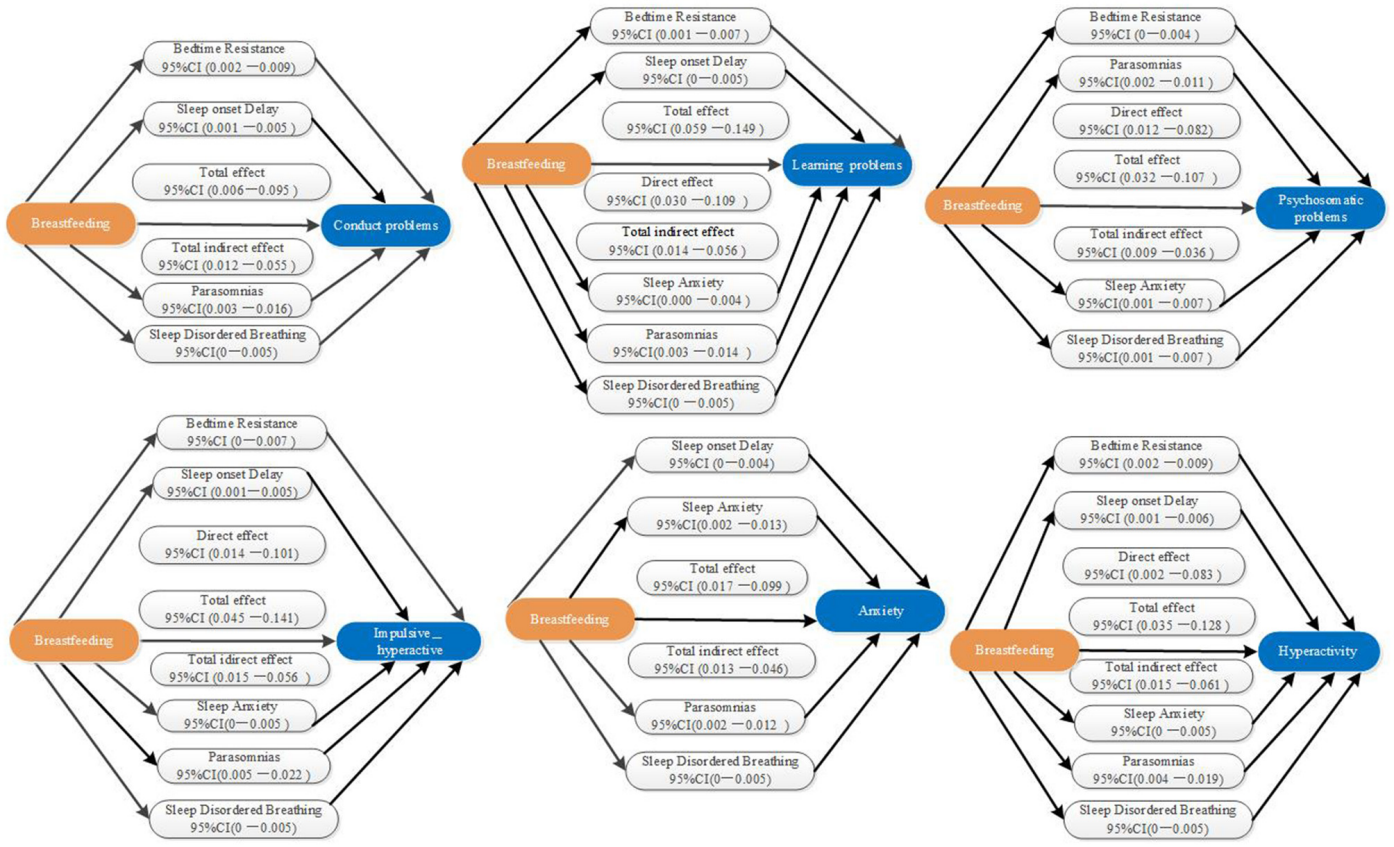
The role of CSHQ dimensions (M) as mediators in the effect of breastfeeding (X) on CPRS behavioral dimensions (Y).

Breastfeeding exhibits a protective effect against behavioral issues partly through its influence on sleep quality. Breast milk contains antimicrobial and anti-inflammatory substances that promote the development of an infant's immature simmune system (26, 27). Exclusively breastfed children show a reduced risk of respiratory infections, which can lead to chronic inflammation and hypertrophy of upper respiratory tract tissues, potentially causing SDB. This study reveals that the sub-item of sleep-disordered breathing in the CSHQ consistently mediates the analysis between breastfeeding and childhood behavioral problems, which is highly consistent with the airway remodeling theory: early respiratory infections in non-breastfed children may lead to chronic hyperplasia of lymphatic tissue, resulting in persistent SDB and neurobehavioral sequelae (Figure 1). This finding aligns with previous research indicating that breastfeeding indirectly promotes children's behavioral health by improving sleep quality (28, 29).

Childhood behavior problems, categorized into internalizing and externalizing behaviors, have profound and lasting negative impacts on academic performance, interpersonal relationships, and parent-child relationships (30, 31). Early intervention is crucial, but it faces barriers such as high costs and limited medical resources. Identifying modifiable risk factors, like breastfeeding and sleep disturbances, is essential for prevention and intervention. Our study found that the absence of breastfeeding, compared to breastfeeding, increases the risk of behavioral problems in children through sleep disturbances. This association remained statistically significant after adjusting for various confounding factors. While previous studies have suggested that breastfeeding may not directly affect behavioral problems in school-aged children, they may not have fully considered the mediating role of sleep disturbances (5–7). Our findings provide a new perspective on the relationship between breastfeeding, sleep disturbances, and children's behavioral problems (Table 7, Figure 1).

Healthy sleep is vital for children's development, and sleep disturbances pose risks for behavioral disorders (32, 33). Breastmilk components assist infants in adapting to day-night changes and regulating sleep/wake cycles (34, 35). Our research supports previous findings that the absence of breastfeeding, compared to breastfeeding, is linked to overall sleep disorders in 6–8-year-olds. However, some studies contrast with our results, potentially due to variations in study design and methods (36). Future research should employ both objective and subjective methods and consider additional variables affecting nocturnal sleep.

Early screening and treatment of sleep disorders in children are crucial. Obstructive Sleep Apnea-Hypopnea Syndrome (OSAHS) is a common sleep disorder, and Polysomnography (PSG) is the gold standard for screening (37). Adenotonsillectomy is the first-line treatment, and studies show improved quality of life post-intervention (38). Therefore, active treatment of sleep disorders in children is crucial.

Based on these findings, we recommend incorporating the CSHQ sleep-disordered breathing items (e.g., “snoring ≥3 nights per week”) into routine physical examinations for school-aged children. For children exhibiting sleep-disordered breathing with CPRS scores >2 standard deviations, priority should be given to home sleep apnea testing to diagnose potential OSAHS. Following confirmation, these children should be referred to a multidisciplinary “Breastfeeding-Sleep-Behavior” clinic, where adenoidectomy may simultaneously improve both OSAS and associated behavioral symptoms (38).

### 4.1 Limitations

Our study has several limitations. Firstly, as a cross-sectional study relying on self-reported data, there may be subjective bias. To mitigate this, detailed instructions were provided for completing the questionnaire. Secondly, other covariates such as family income, genetic factors, medical history, developmental delays, nutritional deficiencies, chronic conditions and family support were not considered may affect the observed associations, and the sample was limited to 6–8-year-olds in Shanghai communities, lacking generality. Future research should consider longitudinal studies with random sampling to enhance representativeness. Thirdly, due to the cross-sectional design with single-timepoint data, it's impossible to determine variable sequence and thus distinguish causes from effects. For instance, though sleep disturbances link to children's behavioral problems, it's unclear which comes first or if both are affected by unmeasured factors like family environment.

### 4.2 Strengths

Despite these limitations, our study has a large sample size. Few studies have conducted mediation analysis on the relationship between breastfeeding and children's behavior with regard to sleep disturbances. This study reveals the relationship between breastfeeding, sleep disorders, and behavior in children. These findings may not only provide a basis for early intervention strategies targeting sleep and parenting practices but also offer new perspectives for clinical attention and the management of these issues.

## 5 Conclusion

This cross-sectional study provides sleep disorders significantly mediate the link between lack of breastfeeding and behavior in 6–8-year-old school-aged children. However, findings are limited by data collection biases and the cross-sectional design's inability to confirm causality. Future cohort or intervention studies are needed to explore causal connections among these factors.

## Data Availability

The datasets presented in this article are not readily available because as per IRB application and permission, research data will not be made available in the public. However, the relevant data can be obtained from the authors upon qualified request and with the consent of Shanghai Sixth People's Hospital affiliated to Shanghai Jiao Tong University School of Medicine. Requests to access the datasets should be directed to sunrisingup@163.com.
