# Supplementary material for: The mediating role of sleep disorders in the relationship between breastfeeding and behavioral problems among 6- to 8-year-old children in Shanghai, China
Source: Front Public Health. 2026 Jan 22;13:1610810. doi: 10.3389/fpubh.2025.1610810 (PMC12875280; doi:10.3389/fpubh.2025.1610810)
Supplement: Supplementary file 1 [file Table_1.docx]

**Supplementary TableS1: The Mediating Effect of CSHQ Total Score on Six Dimensions of the Conners Scale in Breastfeeding Infants**

|  | **Structural paths** | **B (SE)** | **95%CI** | **β** | ‌ p | effect proportion (%) |
| --- | --- | --- | --- | --- | --- | --- |
| Conduct_ problems | Total effect | 0.051 (0.023) | 0.006─0.095 | 0.044 | 0.025 | 100.00 |
|  | direct effect | 0.014(0.021) | -0.026─0.055 | 0.013 | 0.483 | 27.45 |
|  | indirect effect | 0.036(0.01*) | 0.016*─0.055* | 0.032 |  | 70.59 |
| Learning_ problems | Total effect | 0.104 (0.023) | 0.059─0.149 | 0.089 | ＜0.001 | 100.00 |
|  | direct effect | 0.067(0.021) | 0.026─0.108 | 0.057 | 0.001 | 64.42 |
|  | indirect effect. | 0.037(0.010*) | 0.018*─0.054* | 0.032 |  | 35.58 |
| Psychosomatic  problems | Total effect | 0.070 (0.019) | 0.032─0.107 | 0.072 | ＜0.001 | 100.00 |
|  | direct effect | 0.047 (0.018) | 0.011─0.082 | 0.048 | 0.010 | 67.14 |
|  | indirect effect | 0.023(0.006*) | 0.011*─0.036* | 0.024 | . | 32.86 |
| Impulsive ─hyperactive | Total effect | 0.093 (0.024) | 0.045─0.141 | 0.074 | ＜0.001 | 100.00 |
|  | direct effect | 0.057 (0.023) | 0.013─0.102 | 0.046 | 0.011 | 61.29 |
|  | indirect effect  _ | 0.036 (0.010*) | 0.015*─0.055* | 0.029 |  | 38.71 |
| Anxiety | Total effect | 0.058 (0.021) | 0.017─0.099 | 0.054 | 0.006 | 100.00 |
|  | direct effect | 0.027 (0.019) | -0.010─0.065 | 0.026 | 0.155 | 46.55 |
|  | indirect effect | 0.030 (0.008*) | 0.014*─0.046* | 0.028 |  | 51.72 |
| Hyperactivity | Total effect | 0.081 (0.024) | 0.035─0.128 | 0.068 | 0.001 | 100.00 |
|  | direct effect | 0.041 (0.021) | 0.000─0.083 | 0.034 | 0.052 | 50.62 |
|  | indirect effect | 0.040 (0.01*) | 0.019*─0.060* | 0.033 |  | 49.38 |

Note: * The confidence interval (CI) for the total direct effect was calculated using the Bootstrap method; CPRS questionnaires: Conners Parent Rating Scale.
